# Supplementary material for: Responsiveness of domesticated goats towards various stressors following long-term cognitive test exposure
Source: PeerJ. 2022 Mar 29;10:e12893. doi: 10.7717/peerj.12893 (PMC8973470; doi:10.7717/peerj.12893)
Supplement: Supplemental Information 2 [file peerj-10-12893-s002.pdf]

Supplementary Information to:

Responsiveness of domesticated goats towards different stressors following long-term cognitive test exposure

ROSENBERGER, K.[1,2*], SIMMLER, M.[3], LANGBEIN, J.[4], NAWROTH, C.[4], KEIL, N.[1*]

[1]Swiss Federal Food Safety and Veterinary Office, Centre for Proper Housing of Ruminants and Pigs, Agroscope, 8355 Ettenhausen, Switzerland

[2]Graduate School for Cellular and Biomedical Sciences, University of Bern, 3012 Bern, Switzerland

[3]Digital Production, Agroscope, 8355 Ettenhausen, Switzerland

[4]Institute of Behavioural Physiology, Research Institute for Farm Animal Biology, 18196 Dummerstorf, Germany

*Corresponding authors:

Katrina Rosenberger

Agroscope, Tänikon 1, 8356 Ettenhausen, Switzerland

katrinarosenberger91@gmail.com

Nina Keil

Agroscope, Tänikon 1, 8356 Ettenhausen, Switzerland

nina.keil@agroscope.admin.ch

Description of the habituation to the conditions for treatment administration

Prior to administration of the treatments (COG, POS, ISO; Table S1), all goats underwent individual shaping, training, and habituation to the conditions of the later treatment administration. Therefore, they were physically and visually separated from their pen-mates in the room (450 cm x 200 cm) in which later the treatments were administered, but had acoustic contact to their pen-mates that were located in an adjacent waiting area (600 cm x 200 cm). An experimenter sat in an adjacent compartment (150 cm x 200 cm) separated from the animal by a grate, through which the animal could put its snout. A sliding board (60 cm x 20 cm) was placed on the experimenter side of the grate on a small table (105 cm x 40 cm) at a height of approximately 35 and 40 cm for dwarf goats and dairy goats, respectively.

All animals were habituated at first in the whole group of a pen (2 d; 20 min per day), then in groups of two (4–6 d; 5 min per day), and finally individually (7–13 d; 2 min per day). For the goats which later received the COG and POS treatment, the habituation continued until each animal confidently took a reward (a piece of uncooked pasta) from the sliding board. Only the future COG goats continued with the following 'shaping' phase (10 trials per session) in which the animals were trained to indicate a choice. A plant saucer (2 cm high, Ø 14 cm) with a reward was placed in the middle of the sliding board (first 4 trials of each 'shaping' session). As soon as the animal stuck its nose through the middle of the grate, it received the reward from the experimenter. In the next step, the reward was covered with a cup (plant pot, light brown, 10.5 cm high, Ø 12 cm) before the animal could make a choice (last 6 trials of each 'shaping' session). The goal of the 'shaping' phase was achieved when the animals showed no signs of stress during the manipulation of and feeding from the sliding board. This phase was followed by further training sessions of 10 trials, in which 2 saucers with cups were positioned on the left and right side of the sliding board (30 cm distance). The experimenter baited, visible to the goat, one of the saucers and then covered both saucers with cups. The goat received the reward only if it chose the baited cup. The location (left or right) of the rewarded cup was pseudo-randomised. The training criterion required that the goat chose the baited side in at least 8 out of 10 trials over two consecutive sessions to proceed to the cognitive tests.

Cognitive tests (COG treatment)

In 44 test sessions distributed over a period of 4–5 months, goats from the COG group were exposed to cognitive tests in the form of object-choice tasks to assess their discrimination and reversal learning skills (1st phase, see Supplementary Table S1) as well as their ability to use physical cues (2nd phase, five clues plus control condition) and human gestures (3rd phase, five gestures plus control condition) to locate a hidden reward in a cognitive test battery.

Table S1: Detailed description of handling and cognitive tests used in the COG, POS, and ISO treatment.

Treatment	Assessed cognitive skill	Task description	Rewards	Time isolated in test arena	Sessions (1 session per day)
COG 1st phase	Visual Discrimination	Object choice between a black and a white cup, with always only one colour of the cups baited.	Dependent on number of trials solved, but a max. of 14 (=12 trials plus 2 motivational trials*)	Until all trials solved or a max. of 10 min	20 sessions of 12 trials
	Reversal Learning	Object choice task with reversed cup colours, with always only one colour of the cups baited.			
COG 2nd phase	Visual exclusion using direct information	Choice between a baited cup and an unbaited cup, which are both lifted, providing direct visual information regarding the location of a food reward	Dependent on number of trials solved, but a max. of 14 (=12 trials plus 2 motivational trials*)	Until all trials solved or a max. of 10 min	12 sessions of 12 trials
	Visual exclusion using indirect information	Choice between a baited cup and an unbaited cup, where only the empty cup is lifted providing indirect visual information regarding the location of a food reward			
	Auditory exclusion using direct information	Choice between a baited cup and an unbaited cup, both lifted and shaken, providing direct auditory information regarding the location of a food reward			
	Auditory exclusion using indirect information	Choice between a baited cup with food and an unbaited cup, both lifted but only one shaken, providing indirect auditory information regarding the location of a food reward			
	Transposition	Of two cups, one is baited in full view of the animal before the other cup is moved to the right side and the right cup to the left side			
COG 3rd phase	Using sustained pointing gesture	The experimenter is positioned in between two cups, pointing at the baited cup until the animal makes a choice	Dependent on number of trials solved, but a max. of 14 (=12 trials plus 2 motivational trials*)	Until all trials solved or a max. of 10 min	12 sessions of 12 trials
	Using momentary pointing gesture	The experimenter is positioned in between two cups, pointing at the baited cup for about one second			

	Using pointing vs. body orientation	The experimenter is positioned behind the unbaited cup and points with ipsilateral arm and finger at the baited cup			
	Using body orientation	The experimenter is positioned in between two cups and orients body and head towards the baited cup			
	Using a marker	The experimenter is positioned in between two cups and places a marker on top of the baited cup			
POS	NA	NA	median number of rewards received by COG group in the previous test session	median time taken by COG group to finish all trials in the previous test session	20 sessions
ISO	NA	NA	No rewards	median time taken by COG group to finish all trials in the previous test session	20 sessions

*To ensure motivation prior to each session, subjects received 2 motivational trials in which the reward was visibly placed on a saucer that could be reached by putting the snout through the grate.

Table S2: LMER model and random effect variance components for the rotated component 'active' in the novel arena test (NA_RC1). Abbreviations: est = estimate, s.e. = standard error, sd = standard deviation, z = z-score

**Fixed effects**	*Dwarf*				*Dairy*			
	est.	*s.e.*	*z*	*p(>|z|)*	*est.*	*s.e.*	*z*	*p(>|z|)*
COG	-0.11	0.52	-0.22	0.83	-0.24	0.50	-0.47	0.64
POS	0.32	0.52	0.60	0.55	-0.23	0.50	-0.46	0.65
ISO	-0.11	0.51	-0.21	0.83	0.13	0.50	0.27	0.79

**Random effects**	*variance*	*sd*	*n groups*
Pen	0.13	0.36	12
Site	0.37	0.61	2
Residual	0.73	0.85	87

**Treament contrasts**	*Dwarf*				*Dairy*			
	est.	*s.e.*	*z*	*p(>|z|)*	*est*	*s.e.*	*z*	*p(>|z|)*
POS - COG	0.43	0.35	1.21	0.23	0.01	0.31	0.03	0.98
ISO - POS	-0.42	0.34	-1.24	0.22	0.36	0.30	1.22	0.22
ISO - COG	0.00	0.32	0.01	0.99	0.37	0.30	1.23	0.22

**Selection line contrasts**	*COG*				*POS*				*ISO*			
	est.	*s.e.*	*z*	*p(>|z|)*	*est.*	*s.e.*	*z*	*p(>|z|)*	*est.*	*s.e.*	*z*	*p(>|z|)*
Dairy-Dwarf	-0.13	0.39	-0.32	0.75	-0.55	0.39	-1.38	0.17	0.24	0.37	0.65	0.52

Table S3: LMER model and random effect variance components for the rotated component 'reactive to isolation' in the novel arena test, NA_RC2. (est = estimate, s.e. = standard error, sd = standard deviation, z = z-score)

**Fixed effects**	*Dwarf*				*Dairy*			
	est.	*s.e.*	*z*	*p(>|z|)*	*est.*	*s.e.*	*z*	*p(>|z|)*
COG	-0.54	0.28	-1.91	0.06	0.49	0.26	1.87	0.06
POS	-0.39	0.29	-1.34	0.18	0.48	0.26	1.86	0.06
ISO	-0.91	0.27	-3.35	<0.001	0.48	0.25	1.87	0.06

**Random effects**	*variance*	*sd*	*n groups*
Pen	0.20	0.44	12
Site	0.00	0.00	2
Residual	0.55	0.74	87

**Treament contrasts**	*Dwarf*				*Dairy*			
	est.	*s.e.*	*z*	*p(>|z|)*	*est*	*s.e.*	*z*	*p(>|z|)*
POS - COG	0.15	0.31	0.49	0.62	-0.01	0.27	-0.05	0.96
ISO - POS	-0.52	0.30	-1.76	0.08	0.00	0.26	-0.01	0.99
ISO - COG	-0.37	0.28	-1.32	0.19	-0.02	0.26	-0.06	0.95

**Selection line contrasts**	*COG*				*POS*				*ISO*			
	est.	*s.e.*	*z*	*p(>|z|)*	*est.*	*s.e.*	*z*	*p(>|z|)*	*est.*	*s.e.*	*z*	*p(>|z|)*
Dairy-Dwarf	1.03	0.39	2.67	<0.01	0.87	0.39	2.24	0.03	1.38	0.37	3.72	<0.001

Table S4: LMER model and random effect variance components for the rotated component 'active' in the novel object test, NO_RC1. (est = estimate, s.e. = standard error, s.d.=standard deviation, z = z-score)

**Fixed effects**	*Dwarf*				*Dairy*			
	est.	*s.e.*	*z*	*p(>|z|)*	*est.*	*s.e.*	*z*	*p(>|z|)*
COG	0.38	0.35	1.07	0.28	-0.36	0.33	-1.11	0.27
POS	0.55	0.37	1.47	0.14	-0.53	0.33	-1.61	0.11
ISO	0.38	0.33	1.16	0.25	-0.20	0.32	-0.61	0.54

**Random effects**	*variance*	*sd*	*n groups*
Pen	0.05	0.22	12
Site	0.10	0.32	2
Residual	0.78	0.88	89

**Treament contrasts**	*Dwarf*				*Dairy*			
	est.	*s.e.*	*z*	*p(>|z|)*	*est*	*s.e.*	*z*	*p(>|z|)*
POS - COG	0.17	0.38	0.44	0.66	-0.17	0.31	-0.56	0.58
ISO - POS	-0.16	0.36	-0.46	0.64	0.34	0.30	1.10	0.27
ISO - COG	0.00	0.34	0.01	0.99	0.16	0.30	0.55	0.58

**Selection line contrasts**	*COG*				*POS*				*ISO*			
	est.	*s.e.*	*z*	*p(>|z|)*	*est.*	*s.e.*	*z*	*p(>|z|)*	*est.*	*s.e.*	*z*	*p(>|z|)*
Dairy-Dwarf	-0.74	0.36	-2.08	0.04	-1.08	0.38	-2.85	<0.01	-0.58	0.33	-1.77	0.08

Table S5: LMER model and random effect variance components for the rotated component 'exploratory' in the novel object test, NO_RC2. (est = estimate, s.e. = standard error, sd = standard deviation, z = z-score)

**Fixed effects**	*Dwarf*				*Dairy*			
	est.	*s.e.*	*z*	*p(>|z|)*	*est.*	*s.e.*	*z*	*p(>|z|)*
COG	-0.13	0.37	-0.35	0.72	0.13	0.34	0.37	0.71
POS	0.17	0.39	0.45	0.66	0.29	0.35	0.84	0.40
ISO	-0.62	0.35	-1.79	0.07	0.31	0.34	0.92	0.36

**Random effects**	*variance*	*sd*	*n groups*
Pen	0.00	0.00	12
Site	0.13	0.36	2
Residual	0.87	0.93	89

**Treament contrasts**	*Dwarf*				*Dairy*			
	est.	*s.e.*	*z*	*p(>|z|)*	*est*	*s.e.*	*z*	*p(>|z|)*
POS - COG	0.31	0.40	0.77	0.44	0.17	0.32	0.51	0.61
ISO - POS	-0.80	0.38	-2.12	0.03	0.02	0.32	0.06	0.96
ISO - COG	-0.49	0.36	-1.38	0.17	0.18	0.32	0.59	0.56

**Selection line contrasts**	*COG*				*POS*				*ISO*			
	est.	*s.e.*	*z*	*p(>|z|)*	*est.*	*s.e.*	*z*	*p(>|z|)*	*est.*	*s.e.*	*z*	*p(>|z|)*
Dairy-Dwarf	0.26	0.35	0.73	0.46	0.12	0.38	0.31	0.75	0.93	0.32	2.91	<0.01

Table S6: LMER model and random effect variance components for the rotated component ‘sociable’ towards a novel human” in the novel human test, NH_RC1. (est = estimate, s.e. = standard error, sd = standard deviation, z = z-score)

**Fixed effects**	*Dwarf*				*Dairy*			
	est.	*s.e.*	*z*	*p(>|z|)*	*est.*	*s.e.*	*z*	*p(>|z|)*
COG	-0.49	0.70	-0.70	0.48	0.14	0.69	0.21	0.84
POS	-0.08	0.70	-0.11	0.91	0.12	0.69	0.17	0.87
ISO	-0.51	0.69	-0.74	0.46	0.27	0.69	0.39	0.70

**Random effects**	*variance*	*sd*	*n groups*
Pen	0.02	0.15	12
Site	0.89	0.94	2
Residual	0.47	0.69	89

**Treament contrasts**	*Dwarf*				*Dairy*			
	est.	*s.e.*	*z*	*p(>|z|)*	*est*	*s.e.*	*z*	*p(>|z|)*
POS - COG	0.41	0.27	1.53	0.13	-0.03	0.25	-0.11	0.92
ISO - POS	-0.44	0.26	-1.70	0.09	0.15	0.25	0.60	0.55
ISO - COG	-0.02	0.26	-0.08	0.93	0.12	0.24	0.52	0.61

**Selection line contrasts**	*COG*				*POS*				*ISO*			
	est.	*s.e.*	*z*	*p(>|z|)*	*est.*	*s.e.*	*z*	*p(>|z|)*	*est.*	*s.e.*	*z*	*p(>|z|)*
Dairy-Dwarf	0.63	0.27	2.33	0.02	0.19	0.28	0.69	0.49	0.78	0.25	3.05	<0.01

Table S7: LMER model and random effect variance components for the rotated component 'active' in the novel human test, NH_-RC2. (est = estimate, s.e. = standard error, sd = standard deviation, z = z-score)

**Fixed effects**	*Dwarf*				*Dairy*			
	est.	*s.e.*	*z*	*p(>|z|)*	*est.*	*s.e.*	*z*	*p(>|z|)*
COG	0.32	0.38	0.84	0.40	-0.33	0.37	-0.88	0.38
POS	0.64	0.38	1.66	0.10	-0.71	0.38	-1.86	0.06
ISO	0.59	0.37	1.60	0.11	-0.60	0.37	-1.63	0.10

**Random effects**	*variance*	*sd*	*n groups*
Pen	0.02	0.14	12
Site	0.19	0.44	2
Residual	0.64	0.80	89

**Treament contrasts**	*Dwarf*				*Dairy*			
	est.	*s.e.*	*z*	*p(>|z|)*	*est*	*s.e.*	*z*	*p(>|z|)*
POS - COG	0.31	0.31	1.00	0.32	-0.38	0.29	-1.29	0.20
ISO - POS	-0.04	0.30	-0.15	0.88	0.10	0.29	0.36	0.72
ISO - COG	0.27	0.30	0.90	0.37	-0.27	0.28	-0.99	0.32

**Selection line contrasts**	*COG*				*POS*				*ISO*			
	est.	*s.e.*	*z*	*p(>|z|)*	*est.*	*s.e.*	*z*	*p(>|z|)*	*est.*	*s.e.*	*z*	*p(>|z|)*
Dairy-Dwarf	-0.65	0.31	-2.11	0.03	-1.34	0.32	-4.22	<0.001	-1.20	0.29	-4.13	<0.001

Table S8: LMER model and random effect variance components for the PCA component 'reactive towards handling' in the weighing test, WH_PC. (est = estimate, s.e. = standard error, sd = standard deviation, z = z-score)

**Fixed effects**	*Dwarf*				*Dairy*			
	est.	*s.e.*	*z*	*p(>|z|)*	*est.*	*s.e.*	*z*	*p(>|z|)*
COG	-0.03	0.54	-0.05	0.96	-0.47	0.54	-0.87	0.38
POS	0.79	0.55	1.43	0.15	0.01	0.54	0.03	0.98
ISO	0.03	0.54	0.06	0.96	0.05	0.53	0.09	0.93

**Random effects**	*variance*	*sd*	*n groups*
Pen	0.07	0.27	12
Site	0.47	0.69	2
Residual	0.64	0.80	94

**Treament contrasts**	*Dwarf*				*Dairy*			
	est.	*s.e.*	*z*	*p(>|z|)*	*est*	*s.e.*	*z*	*p(>|z|)*
POS - COG	0.81	0.31	2.62	<0.01	0.48	0.28	1.73	0.08
ISO - POS	-0.76	0.31	-2.48	0.013	0.03	0.27	0.12	0.91
ISO - COG	0.06	0.29	0.20	0.85	0.52	0.27	1.91	0.06

**Selection line contrasts**	*COG*				*POS*				*ISO*			
	est.	*s.e.*	*z*	*p(>|z|)*	*est.*	*s.e.*	*z*	*p(>|z|)*	*est.*	*s.e.*	*z*	*p(>|z|)*
Dairy-Dwarf	-0.44	0.32	-1.37	0.17	-0.77	0.34	-2.25	0.02	0.02	0.32	0.05	0.96

Table S9: LMER model and random effect variance components for the variable *entering score* in the weighing test. (est = estimate, s.e. = standard error, s.d.=standard deviation, z = z-score)

**Fixed effects**	*Dwarf*				*Dairy*			
	est.	*s.e.*	*z*	*p(>|z|)*	*est.*	*s.e.*	*z*	*p(>|z|)*
COG	0.30	0.26	1.16	0.25	0.00	0.24	0.02	0.98
POS	0.19	0.29	0.64	0.52	-0.16	0.25	-0.64	0.53
ISO	0.04	0.25	0.17	0.87	-0.28	0.24	-1.16	0.25

**Random effects**	*variance*	*sd*	*n groups*
Pen	0.00	0.00	12
Site	0.00	0.00	2
Residual	1.02	1.01	94

**Treament contrasts**	*Dwarf*				*Dairy*			
	est.	*s.e.*	*z*	*p(>|z|)*	*est*	*s.e.*	*z*	*p(>|z|)*
POS - COG	-0.12	0.39	-0.30	0.77	-0.17	0.35	-0.47	0.64
ISO - POS	-0.14	0.38	-0.37	0.71	-0.12	0.35	-0.33	0.74
ISO - COG	-0.26	0.36	-0.72	0.47	-0.28	0.34	-0.82	0.41

**Selection line contrasts**	*COG*				*POS*				*ISO*			
	est.	*s.e.*	*z*	*p(>|z|)*	*est.*	*s.e.*	*z*	*p(>|z|)*	*est.*	*s.e.*	*z*	*p(>|z|)*
Dairy-Dwarf	-0.30	0.36	-0.83	0.41	-0.35	0.38	-0.90	0.37	-0.32	0.35	-0.92	0.36
